# Developing an integrated conceptual framework of *NewWork*-settings: a systematic scoping review

**DOI:** 10.3389/fsoc.2025.1631523

**Published:** 2025-09-30

**Authors:** Anna-Sophia Wawera, Fiona Niebuhr, Sophie Glaser, Carla Rinne, Susanne Voelter-Mahlknecht

**Affiliations:** ^1^Institute of Occupational Medicine, Charité - Universitätsmedizin Berlin, Corporate Member of Freie Universität Berlin and Humboldt Universität zu Berlin, Berlin, Germany; ^2^Institute of Occupational, Social und Preventive Medicine, University Medical Center, Georg August University, Göttingen, Germany

**Keywords:** modern work, new ways of working (NWOW), work flexibility, digitalization, agility, democratic workplaces

## Abstract

**Introduction:**

The last few decades have seen drastic changes in the world of work. These global transformations of work towards more digital, decentralized and democratic forms are commonly referred to as *NewWork* (NW). NW is frequently described as a container term encompassing a broad set of concepts. In order to provide conceptual clarity, the aim of this review was to develop an integrated conceptual framework of NW-settings, i.e., workplaces that have implemented different NW measures.

**Methods:**

A systematic scoping review was conducted, following the framework by Arksey and O’Malley and the Joanna Briggs Institute.

**Results:**

Based on the synthesis of 99 included articles, we developed a multidimensional conceptual framework of NW-settings, which consists of four dimensions: *flexibility, digitalization, democratization and agility*, and 14 inter-related sub-dimensions.

**Discussion:**

This framework facilitates a clearer understanding of NW and provides valuable insights for contemporary work practices and the broader social implications of digital and organizational transformations in our world of work.

## 1 Introduction

The last few decades have seen drastic changes in the work environment, driven by globalization, digitalization, demographic shifts, and accelerated innovation cycles. These changes have created a growing demand for adaptability in organizational work processes (Kanten, 2014). In addition, advances in information and communication technology (ICT) have significantly contributed to how people communicate and exchange information in the workplace (Kotera and Correa Vione, 2020). At the same time, the global marketplace demands companies to be more responsive towards changes than ever before (Salmen and Festing, 2021). This trend has led to an increase in flexible work arrangements, with the COVID-19 pandemic accelerating this shift, where flexible work has become a prevailing working model for a large part of the population (Ekpanyaskul and Padungtod, 2021). These global transformations of work towards more digital, decentralized and democratic forms are commonly referred to as *NewWork* (NW). The term NW is primarily used in German-speaking regions, while international literature also refers to it as New Ways of Working (NWOW), modern work or new work arrangements. However, due to the absence of a standardized definition, there is no clear conceptual distinction between these terms. Throughout this article, the term NW will be used.

NW is frequently described as a *container term* encompassing a broad and evolving set of concepts (Schermuly, 2024). The origins of NW can be traced back to its founder, the German philosopher Fritjof Bergmann, who conceptualized work as a means of self-determination and personal fulfilment rather than merely a tool for economic productivity (Bergmann, 2017). From a more practical and application-oriented perspective, the term NW refers to a broad set of approaches and strategies that organizations use to restructure their processes and systems in response to changing demands and environments (Schneider, 2020). Thus, NW encompasses a diverse range of concepts, including varied work structures (e.g., agile, holocratic), workplace arrangements (e.g., remote work, working from home), evolving employee capacities and values (e.g., autonomy, resilience), and shifting organizational paradigms (e.g., employee-centric culture, transformational leadership) (Schneider, 2020). Due to the complexity and broad scope of NW itself, however, we decided to focus this scoping review on the narrower concept of *NW-settings*, i.e., workplaces that have implemented different NW-measures. By narrowing our focus to NW-settings, we aimed to focus on the environmental context and circumstances where NW-measures are applied and experienced by employees, reflecting modern work structures, designs, and conditions.

Understanding NW-settings is crucial for both researchers and practitioners seeking to navigate the evolving world of work. For researchers, this review provides conceptual clarity in a field characterized by terminological inconsistencies and a lack of uniform definitions. For practitioners, particularly in human resources (HR), occupational health management (OHM), and organizational development, it offers evidence-based insights into how NW-settings function in practice and what implications they have for employee well-being and organizational performance. By bridging theoretical discourses and practical application, this scoping review aims to support the development of more responsive work environments aligned with the principles of NW.

This scoping review was part of the larger *BGM4NewWork* project at a large university hospital in Germany, which investigated the health benefits and potential risks associated with NW. The goal of this project was to adapt OHM strategies to the specific needs of employees working in NW-settings. Given the conceptual ambiguity surrounding NW, a key initial step in the *BGM4NewWork* project was to first define and operationalize NW-settings. This article presents the findings of a comprehensive scoping review aimed at synthesizing the existing literature on NW-settings. The primary objective of this review was to develop an integrated conceptual framework for understanding NW-settings by synthesizing the currently fragmented literature on NW across different disciplines, with a particular focus on the German context. Thus, this review aims to provide a conceptual framework to enhance clarity on how NW-settings are defined, operationalized, and experienced within specific organizational environments.

## 2 Methods

Considering the complex nature of NW and NW-settings as well as the wide range of existing literature, a systematic scoping review was conducted to provide an overview of the most common forms of NW-settings discussed in the literature. Compared to systematic reviews, which are often considered the gold standard of literature reviews but aim to answer more specific research questions, scoping reviews offer a broader approach to delineating the multi-dimensional aspects of a complex concept and provide a comprehensive overview of different methodological approaches including a broader range of study types (Peters et al., 2015b). Thus, they are used to clarify key concepts and definitions underpinning the research area, to capture the key factors, characteristics and limitations of such concepts (Munn et al., 2018), and to identify current gaps in the literature that can justify and develop future research inquiries (Peters et al., 2015b).

In order to ensure rigour and a systematic approach, this review was guided by the methodological framework by Arksey and O'malley (2005) for conducting scoping reviews, which is most widely used for scoping studies and has been further refined in recent years (e.g., Westphaln et al., 2021). Arksey and O'malley’s (2005) framework outlines six important steps for systematically conducting scoping reviews in order to (1) identify the research questions, (2) identify relevant studies, (3) select studies, (4) chart the data, (5) collate and summarize the data and (6) consult with relevant stakeholders. In addition, the review followed the guidelines by the Joanna Briggs Institute (JBI) on how to conduct a scoping review (Peters et al., 2015a), which have been integrated into the six stages recommended by Arksey and O'malley (2005). This review was built on the recommendations of both frameworks to ensure a highly systematic and transparent approach.

### 2.1 Identifying the research question

The first step of conducting a scoping review is to identify a broad but clear review question. The present scoping review was guided by the following research question: *What NW-settings can be identified in the current literature?* Considering the broad nature of review questions scoping reviews aim to answer, Peters et al. (2015a) recommend the development of wider inclusion criteria, based on the PCC (Population, Concept and Context) framework (Peters et al., 2015b) (see Table 1). The PCC framework for this scoping review was developed based on previous projects and knowledge about NW by the research team, as well as key stakeholders advising the project team (see section “Consulting with relevant stakeholders” below).

**Table 1:** PCC framework.

PCC framework	Included concepts
Population	Employees and employers working in NW-settings
Concept	Different forms of NW: e.g. agility, flexibility, empowerment, work-life-balance, leadership, participation, digitalization, autonomy
Context	English and German publications, published in the last 10 years given rapid changes in the work environment

### 2.2 Identifying relevant studies

To identify relevant published articles, six key electronic social science, occupational health and psychology databases were searched: MEDLINE, PsycArticle, PsycInfo, Psyndex, PubMed and Web of Science. In addition, the reference lists of included articles and relevant reviews were searched. The initial search was completed in October 2022 and updated in May 2024. Due to the deliberately broad research question as well as economic aspects, gray literature was not extracted.

Key search terms were identified based on the PCC framework introduced above, using MeSH terms, free-text and thesaurus searching, as well as looking for appropriate search terms in related literature reviews. Table 2 shows the search terms that were used across the databases and how they were combined using Boolean operators ‘OR’ and ‘AND’ within and across concepts.

**Table 2:** Overview of search terms.

N	Concepts	Search terms
1	NewWork	“New work” OR “work 4.0”
AND		
2	Agility	Agility OR agile OR “agile development” OR “agile framework” OR “Workforce agility” OR “organisational agility” OR “non-hierarchical” OR “self-organised” OR “agile organisation”
OR		
3	Flexibility	Flexibility OR “Work flexibility” OR “workplace flexibility” OR flex OR “remote work*” OR “home office” OR “telework*” OR “virtual work” OR “working from home” OR “blended working” OR telework* OR “hybrid work”*
OR		
4	Work-Life-Balance	“Work-life conflict” OR “work-family balance” OR “work–family conflict” OR “quality of work life”
OR		
5	Digitalization	“Digitalization” OR “digitalization” OR “digital transformation” OR “digital technology” OR “online” OR “digital competency”
OR		
6	Digital Leadership	“Digital leadership” OR “Holocracy” OR “digital transformation leadership”
OR		
7	Connectivity	“Connectivity” OR “network”
OR		
8	Autonomy	Autonomy OR independence OR self-determination OR meaningfulness OR coherence OR empowerment
OR		
9	Participation	Participation OR engagement OR involvement

Based on the PCC framework, the review included studies that met the following inclusion and exclusion criteria (Table 3).

**Table 3:** Inclusion and exclusion criteria.

Inclusion criteria	Exclusion criteria
Articles focusing on different forms of NW Journal articles Book chapters Publications in German and English Publications from January 2012 to January 2024 Theoretical and primary research, such as qualitative, quantitative and mixed-method studies, as well as relevant literature reviews	Articles focusing on traditional forms of work Publications before 2012 and not in English or German Full-texts not available Books Dissertations Gray literature

### 2.3 Study selection

**Figure 1:**
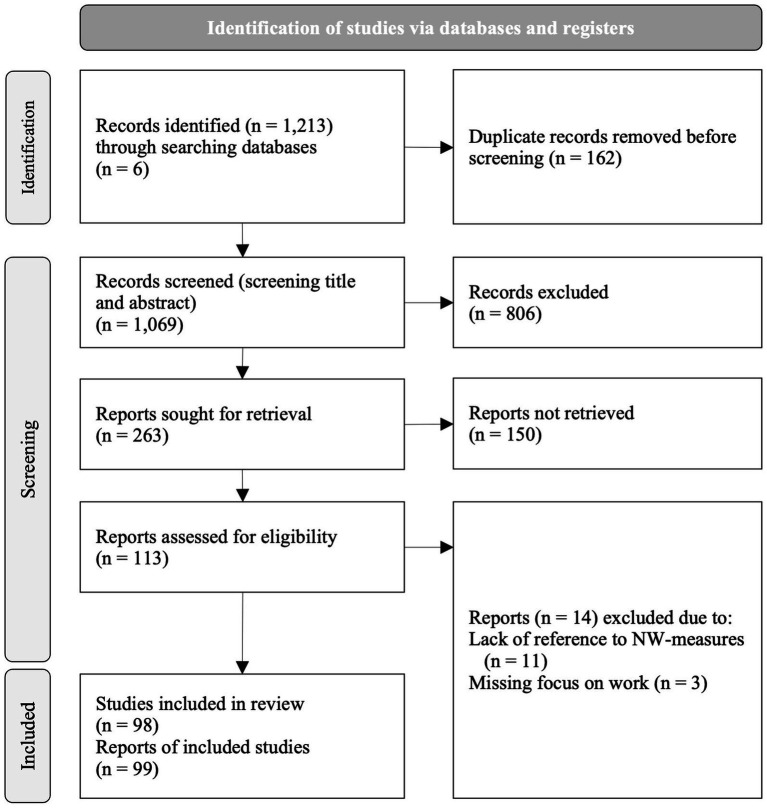
PRISMA flow diagram.

As Figure 1 illustrates, the searches identified a total of 1,213 articles from the six databases. All articles were imported into the electronic data management system EndNote 21. After removal of duplicates (*n = 162*), titles and abstracts of articles were screened (*n = 1,069*). Articles where the title and abstract were relevant to the review question and met the inclusion criteria were obtained in full text (*n = 113*). After reading the full-text articles, further 14 articles were excluded, as they did not sufficiently discuss any NW-measures or did not focus on work settings. In total, 99 articles based on 98 studies [the articles Ekpanyaskul et al. (2023) and Ekpanyaskul and Padungtod (2021) were based on the same study] were included in this review.

### 2.4 Charting the data

Next, general and data specific to the review question were extracted from the included studies and transferred to a data extraction form in Excel. Following Arksey and O'malley’s (2005) guidelines, data were extracted for authors, year of publication, title, type of publication, country, study population, methods, data collection and analysis, forms of NW-settings, as well as strength and limitations of the included article.

### 2.5 Collating, summarizing and reporting the results

For the general characteristics of the included studies, numerical and narrative description of findings were used to summarize the results, following Arksey and O'malley (2005). The different forms of NW-settings were extracted from the literature into a qualitative analytics tool MAXQDA and analyzed using thematic analysis by Braun and Clarke (2021). The major themes in relation to NW-settings identified in the included literature were then mapped out in a diagrammatic format and summarized in narrative form for each theme (Arksey and O'malley, 2005; Peters et al., 2015b). The identified themes formed the four key dimensions of the integrated conceptual framework of NW-settings, which will be presented in detail in the results section.

### 2.6 Consulting with relevant stakeholders

A key component of this systematic scoping review was the active involvement of relevant stakeholders in developing the integrated conceptual framework of NW-settings. Throughout the different stages of the scoping review process, a diverse range of stakeholders contributed their expertise. These included various experts providing advice to companies in NW-settings, employer associations, industry experts, as well as representatives from health insurance providers and occupational accident insurance institutions. The review was informed by the expert knowledge gathered through a series of workshops. In the initial workshop, stakeholder insights were used to identify relevant search terms for the scoping review. Subsequently, a second workshop facilitated the development and refinement of the integrated conceptual framework of NW-settings.

## 3 Results

### 3.1 Characteristics of included articles

Overall, a total of 99 articles from 98 studies were included in the review. The articles by Ekpanyaskul et al. (2023) and Ekpanyaskul and Padungtod (2021) reported about the same study. Supplementary Table 1 provides an overview of the 99 included articles. The review encompassed various study designs, with 32 quantitative studies, 20 qualitative studies, 12 mixed-method approaches, 13 reviews, and 21 overviews. Most articles explored the perspective of employees/workers (*n = 20 articles*), particularly remote workers (*n = 11*) and digital workers (*n = 10*), as well as the perspective of employers/managers (*n = 19*). Overall, all industries have been explored in the literature, with a focus on manufacturing (*n = 15*), banking and insurance (*n = 14*), IT (*n = 13*) and the public sector (*n = 13*). Geographically, the majority of studies originated from German-speaking countries (*n = 53*), followed by other European countries (*n = 26*) and North America (*n = 11*).

### 3.2 Introduction of the integrated conceptual NW-settings framework

Based on the thematic analysis of the included articles (Braun and Clarke, 2021), we found that NW-settings appeared to comprise of four key dimensions: flexibility, digitalization, democratization, and agility. Table 4 below provides an overview of which articles discussed which key dimension, highlighting that the dimension of flexibility was discussed most frequently in the included articles (*n = 40*), followed by democratization (*n = 34*), digitalization (*n = 31*), and agility (*n = 16*).

**Table 4:** Overview of synthesized NW-settings.

NW-setting dimensions		
Flexibility	40	Adolph et al. (2016), Afota et al. (2024), Aroles et al. (2019), Baba et al. (2021), Barbour et al. (2024), Becker et al. (2020), Bender et al. (2022), Braml (2022), Caldeira et al. (2023), Camp et al. (2022), Chen et al. (2023), Degen and Zekavat (2022), Edsall and Conrad (2021), Ekpanyaskul and Padungtod (2021), Ekpanyaskul et al. (2023), Figueira and da Costa (2022), Geldart (2022), Graßmann and Decius (2023), Hardering (2021), Houghton et al. (2018), Khanwalkar and Dabir (2022), Kinsman et al. (2024), Klaser et al. (2023), Klinksiek et al. (2023), Kortsch et al. (2022), Kossek and Ollier-Malaterre (2020), Niebuhr et al. (2022), Nicolaisen (2014), Parent-Lamarche and Laforce (2022), Parker and Knight (2024), Petrakova et al. (2021), Poethke et al. (2019), Silva Júnior et al. (2022), Smite et al. (2023), Soubelet-Fagoaga et al. (2021), Stoian et al. (2022), Terry (2022), Varma et al. (2022), Wong et al. (2020), Zamani and Spanaki (2023)
Digitalization	31	Adolph et al. (2016), Afota et al. (2024), Ackermann et al. (2021), Berretta et al. (2023), Fregnan et al. (2022), Fuchs and Cumbers (2023), Georg et al. (2017), Georgi (2021), Graßmann and Decius (2023), Hardering (2021), Ivaldi et al. (2022), Jäckel (2020), Jochmaring and York (2023), Kortsch et al. (2022), Kuzior et al. (2022), Labanauskaitė et al. (2021), Lin and Wang (2022), Madsen (2019), Ötting et al. (2021), Popescu et al. (2020), Poethke et al. (2019), Rangraz and Pareto (2021), Rohwer et al. (2020), Santa and Popescu (2021), Schermuly and Koch (2019), Schlie and Wendland (2023), Schneider (2020), Schweitzer et al. (2020), Stegh and Guthier (2021), Strikovic and Wittmann (2022), von Garrel and Düben (2022)
Democratization	34	Akin and Rumpf (2013), Aroles et al. (2019), Arzenšek et al. (2021), Bayo-Moriones et al. (2015), Biemann and Weckmüller (2015), Böhm and Stiglbauer (2019), Braml (2022), Chen et al. (2023), Christensen (2023), Claassen et al. (2021), Degen and Zekavat (2022), Deng and Joshi (2016), Dolce et al. (2020), Florin and Pichault (2020), Georg et al. (2017), Gouda and Tiwari (2024), Graßmann and Decius (2023), Göllner and Rau (2021), Kästner and Rudolph (2022), Kesselmann and Böhnke (2021), Kühn et al. (2019), Lin and Wang (2022), Parker and Knight (2024), Poethke et al. (2019), Rohwer et al. (2020), Rožman et al. (2023), Schermuly (2019), Schermuly and Koch (2019), Schmitz et al. (2021), Scholl (2020), Schölmerich et al. (2023), Stecker and Kionke (2020), von Garrel and Düben (2022), Zamani and Spanaki (2023), Zirkler (2023)
Agility	16	Ackermann et al. (2021), Barth and Blazejewski (2023), Bachmann (2022), Bachmann and Quispe Bravo (2021), Coban and Wenten (2021), Confal et al. (2021), Fregnan et al. (2022), Hasenbein (2021), Ivaldi et al. (2022), Kreyenberg (2023), Parent-Lamarche and Laforce (2022), Rödel and Krach (2023), Salmen and Festing (2021), Schneider (2020), Singe and Tietel (2019), Wendt (2023)

**Figure 2:**
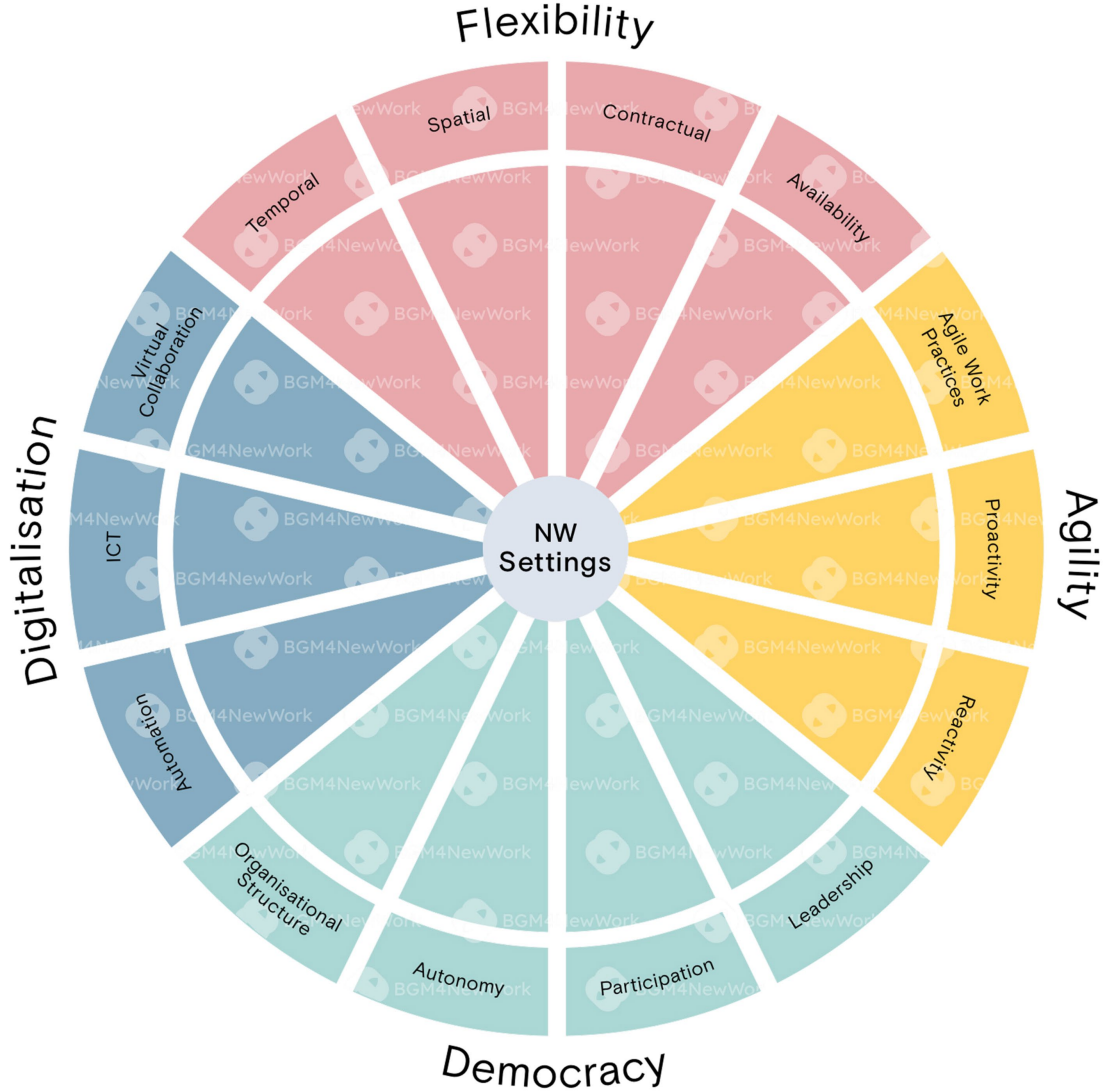
Integrated conceptual framework of NW-settings.

Each dimension was then further divided into 14 interrelated sub-dimensions. Figure 2 illustrates the complete integrated conceptual framework of NW-settings. It is important to note, however, that these dimensions and sub-dimensions often overlapped and did not have clear conceptual boundaries. For example, spatial and temporal flexibility (two sub-dimensions of flexibility) are enabled by the increasing use and development of ICTs and virtual collaboration (sub-dimensions of digitalization). When implemented effectively, these factors can enhance employees’ sense of autonomy and participation at work (two sub-dimensions of democratization). Additionally, our findings suggest that the significance of the four dimensions appeared to exist along a continuum, i.e., a workplace may implement NW-measures across all four dimensions or focus on just one sub-dimension and still be considered a NW-setting.

In the next section, the key dimensions and their sub-dimensions will be discussed in more detail, summarizing key components of each dimension, as well as common advantages and disadvantages discussed in the literature.

#### 3.2.1 Flexibility

The dimension that was discussed the most in the included articles was *flexibility* (see Table 4), particularly in relation to *spatial flexibility*, *temporal flexibility*, as well as *contractual flexibility*, all of which appeared to be related to an increased demand of *employee availability*.

Most articles defined *flexibility* as work arrangements that do not have a set time and place and that mix different arrangements, such as remote, part-time or project-based work (Aroles et al., 2019; Becker et al., 2020; Bender et al., 2022; Graßmann and Decius, 2023; Klaser et al., 2023; Klinksiek et al., 2023; Kortsch et al., 2022; Kuzior et al., 2022; Labanauskaitė et al., 2021). As a result, articles discussed how flexibility offers employees the freedom to choose where, when and how they wish to work (Klinksiek et al., 2023). However, some articles used the term in a broader sense and included a wide range of operational measures which aim to break down rigid and established structures of a company in order to become more flexible (Barth and Blazejewski, 2023; Bender et al., 2021). According to Schlie and Wendland (2023), for example, internal operational measures can include flexible working hours, workplace, work environment, work design, and renumeration, while external operational measures in relation to work flexibility tend to focus on recruitment opportunities, such as work placements, different forms of contracts (employment and service contracts), and personnel outsourcing. Consequently, different forms of flexible work arrangements have been discussed in the included articles, such as working from home (WFH) (Braml, 2022; Chen et al., 2023), hybrid work (Braml, 2022), telework (Dolce et al., 2020; Figueira and da Costa, 2022), telecommuting (Figueira and da Costa, 2022), smart work model (combining the opportunities of working remotely and on-site) (Santa and Popescu, 2021), reduced workload as a flexible form of part-time work (Kossek and Ollier-Malaterre, 2020), and blended working (Figueira and da Costa, 2022).

Similar to Aroles et al. (2019) and Poethke et al. (2019), we found it useful to distinguish between three types of flexibility: *spatial flexibility*, *temporal flexibility*, and *contractual flexibility*, which will be discussed in more detail in the following sections.

##### 3.2.1.1 Spatial flexibility

The included literature referred to spatial work flexibility as the independent choice of employees with regards to the place of work. Over time, traditional offices have gradually lost their importance (Ekpanyaskul et al., 2023). The included literature reported that spatial flexibility gives employees the option to carry out their regular work tasks at different locations of their own choosing (Bender et al., 2021; Klaser et al., 2023; Poethke et al., 2019; Zamani and Spanaki, 2023). Within this literature, different types of spatial flexibility have been discussed, of which the most important ones were:

- Remote working was used as the broadest term and referred to as “the ability of employees to work outside the company, usually at home, in a coworking area, in parks, or any other place from which they connect with […] the organization” (Klaser et al., 2023, p. 3)
- Telework was defined as computer-based employees working away from the employer premises (Kinsman et al., 2024).
- Finally, the majority of the included articles discussed spatial work flexibility in relation to working from home (WFH), “a work arrangement in which the work is done from places other than a traditional office space using information and communication technologies” (Chen et al., 2023, p. 2).

Many articles discussed spatial flexibility in the context of the COVID-19 pandemic, emphasizing how the COVID-19 pandemic acted as a catalyst for and accelerated spatial work flexibility (Camp et al., 2022; Confal et al., 2021; Kesselmann and Böhnke, 2021; Niebuhr et al., 2022; Stoian et al., 2022). Some of the articles also argued that the COVID-19 pandemic ‘forced’ many people to work from home and make those changes rapidly (Barbour et al., 2024; Caldeira et al., 2023; Chen et al., 2023; Kuzior et al., 2022; Parker and Knight, 2024; Smite et al., 2023) often without sufficient preparation or adequate concerns for occupational health and safety (Ekpanyaskul et al., 2023; Parent-Lamarche and Laforce, 2022). With the end of the COVID-19 pandemic, several authors argued that WFH may be a lasting consequence (Chen et al., 2023; Smite et al., 2023), and even though many employees have returned to traditional offices, a hybrid model of work seems to have become the ‘new normal’, with employees working some days in the office and other days at home (Kinsman et al., 2024).

Spatial work flexibility was also discussed in terms of creating new office concepts, ranging from co-working spaces or flexible work centers (FWCs) (Aroles et al., 2019; Figueira and da Costa, 2022; Houghton et al., 2018; Kinsman et al., 2024), flex- and multi-space offices, activity-based working (Klinksiek et al., 2023), and hot-desking (Aroles et al., 2019; Houghton et al., 2018; Klinksiek et al., 2023). Such flexible space concepts are generally used by a heterogeneous mix of workers from various organizations, providing them with the opportunity to interact in an otherwise potentially isolating space at home (Kinsman et al., 2024).

Overall, several advantages and challenges have been reported in relation to spatial work flexibility: Due to the high degree of autonomy and level of freedom spatial flexibility offers employees (Barth and Blazejewski, 2023; Georg et al., 2017; Silva Júnior et al., 2022), it has been associated with an increase in the process of self-actualization, motivation, meaningfulness, happiness at work, and commitment (Biemann and Weckmüller, 2015; Kossek and Ollier-Malaterre, 2020; Kortsch et al., 2022; von Garrel and Düben, 2022). Especially in the area of knowledge work, mobile and distributed work have been found to provide advantages over presence time at companies as it offers more freedom and flexibility to employees, reduces travel time, and enables employees to live and work from rural areas, often providing a better work-life balance (Poethke et al., 2019; Schweitzer et al., 2020; von Garrel and Düben, 2022).

On the other hand, articles have reported health related problems in relation to spatial flexibility, including strain on the eye due to high amount of screen-based work (Khanwalkar and Dabir, 2022) and sleep disturbances associated with increased levels of perceived stress (Rohwer et al., 2020). The latter may be a result of greater work intensification, competition, work-on-demand (Labanauskaitė et al., 2021), as well as an increased demand for employee availability associated with ability to work from anywhere (see section 3.2.1.4 Availability) (Akin and Rumpf, 2013; Confal et al., 2021; Rohwer et al., 2020; Stoian et al., 2022; von Garrel and Düben, 2022). In this context, articles also reported the risk of spatial boundaries between work and private life becoming increasingly blurred (Adolph et al., 2016; Georg et al., 2017; von Garrel and Düben, 2022).

##### 3.2.1.2 Temporal flexibility

Temporal flexibility has also become a defining aspect of modern work, which was discussed in the literature as even more relevant than other NW-measures such as remote or hybrid work (Zamani and Spanaki, 2023). According to Nicolaisen (2014), debates on working time regulations have centered on flexibility and deregulation since the 1980s in many Western countries, leading to more adaptable working time schemes. Employees increasingly prefer individual self-regulation, making it difficult for companies to uphold traditional working hours (Nicolaisen, 2014). This shift seems to aligns with broader social changes, including dual-earner households and a 24-h economy, fostering diverse work arrangements such as flextime, which allows employees to allocate their contracted hours more freely (Klinksiek et al., 2023; Zamani and Spanaki, 2023). Kühn et al. (2019) also discussed how the conventional 9-to-5 model is eroding, being replaced by a results-oriented culture that prioritizes performance over physical presence.

On the one hand, flexible work schedules have been found to support work-life balance and employee autonomy (Barth and Blazejewski, 2023; Georg et al., 2017; Silva Júnior et al., 2022), with research highlighting benefits such as increased perceived organizational support and alignment with work design theories that emphasize self-determination (Biemann and Weckmüller, 2015; Kortsch et al., 2022; Kossek and Ollier-Malaterre, 2020; Poethke et al., 2019; von Garrel and Düben, 2022). On the other hand, temporal flexibility also has challenges. Several articles discussed how the ability to work anytime can lead to work intensification and blurred work-life boundaries (Adolph et al., 2016; Georg et al., 2017; Labanauskaitė et al., 2021; von Garrel and Düben, 2022). Florin and Pichault (2020) also argued that companies working with highly flexible temporal work structures tend to transfer the responsibility for skills development to the employee. Von Garrel and Düben (2022) elaborated that work flexibility requires significant employee self-organization and problem-solving skills from employees, as well as good communication, cooperation and coordination within the organization.

##### 3.2.1.3 Contractual flexibility

In addition, the literature discussed the rise of new employment modalities, such as zero-hour contracts, freelancing, and gig work, and how they significantly reshaped traditional employment structures (Aroles et al., 2019). Online labor platforms, for example, continue to grow in popularity alongside crowd-based and collaborative entrepreneurship models (Aroles et al., 2019). Unlike conventional long-term employment, work duration has become more fluid, with an increasing reliance on short-term, temporary, and project-based arrangements. This evolving contractual landscape encompasses direct employment (full-time and part-time roles), co-employment (through agencies), and independent contract work, offering organizations greater flexibility while also introducing uncertainty for workers (Aroles et al., 2019).

While contractual flexibility can empower workers by linking pay to performance (Scholl, 2020) and supporting diverse employment arrangements (Poethke et al., 2019), it has also been associated with precarious labor conditions. Delivery workers, for example, often face short-term contracts, low wages, and weak worker representation (Fuchs and Cumbers, 2023). In Germany, discussions on alternative labor models, such as universal basic income, highlight efforts to mitigate economic instability in flexible work arrangements (Georgi, 2021; Hardering, 2021). However, challenges persist, including discrepancies between agreed and actual working hours, the complexities of flexible scheduling, and the need for mobility and multiple job engagements (Adolph et al., 2016). Other studies have found that contractual flexibility can lead to employees changing workplaces more frequently which can cause a reduction of workplace commitment (Bender et al., 2021). To navigate these shifts, Akin and Rumpf (2013) concluded that companies should rethink employee loyalty beyond traditional contracts. As personal values and work-life balance become increasingly important to workers, fostering commitment will require aligning corporate expectations with individual goals rather than relying solely on contractual ties (Akin and Rumpf, 2013; Bender et al., 2021).

##### 3.2.1.4 Availability

As discussed in the previous sections, spatial, temporal and contractual flexibility have fundamentally reshaped our working life, enabling employees to work anytime and anywhere. While this increased connectivity offers flexibility, the literature also discussed how it fosters work intensification and an “always-on” culture (Labanauskaitė et al., 2021). The ability to reach employees instantly, accelerated by the spread of ICTs (see next section on the dimension of digitalization), creates a culture of immediacy that can lead to blurred boundaries between work and personal life (Adolph et al., 2016; Aroles et al., 2019; Georg et al., 2017; Göllner and Rau, 2021; von Garrel and Düben, 2022). Aroles et al. (2019) also found that while gig and contract workers theoretically have significant schedule flexibility, in practice, many find themselves unable to fully control their work schedules due to client demands and job insecurity. This phenomenon, known as “extended availability,” reflects an unregulated expectation that workers remain accessible beyond their formal working hours, particularly in global organizations that operate across different time zones (Göllner and Rau, 2021).

This extended availability has introduced new challenges, such as increased work intensity, chronic stress and sleep disruptions due to the pressures of a 24-h society (Aroles et al., 2019; Rohwer et al., 2020; von Garrel and Düben, 2022). However, it also presents opportunities. According to Göllner and Rau (2021), workers with high availability often experience greater autonomy, decision-making power, and access to information. Nonetheless, as different forms of flexibility continue to dissolve the boundaries between work and personal life, balancing availability with well-being remains a critical challenge, one that employees and organizations must navigate to maintain a sustainable and healthy work environment (Labanauskaitė et al., 2021; Poethke et al., 2019).

As shown in the previous section, flexibility was closely linked to other NW-settings dimensions, particularly in relation to digitalization, which will be discussed in the next section.

#### 3.2.2 Digitalization

Digitalization was the second NW-setting that was discussed in the included articles. It primarily refers to the use of and increasing reliance on *information and communication technologies (ICTs)* and *virtual collaboration* but has also been discussed in relation to increased *automation,* including the increasing use of AI, forming the three sub-dimensions of digitalization in this conceptual framework.

Digitalization was discussed as the most important driver transforming work life, particularly in relation to optimizing organizational structures and processes (Claassen et al., 2021; Labanauskaitė et al., 2021). Workflows have been improved through digital networking and human computer interaction, while physical boundaries have lost significance (Schlie and Wendland, 2023). In this context, the COVID-19 pandemic has been discussed as an important catalyst for digital transformation (Afota et al., 2024; Fregnan et al., 2022; Gouda and Tiwari, 2024; Kuzior et al., 2022; Niebuhr et al., 2022; Rožman et al., 2023). The pandemic accelerated the adoption of new forms of communication and collaboration, such as video conferencing, and mobile working has become a permanent feature of working life (Jochmaring and York, 2023). Digitalization has also been discussed as an essential component of work 4.0 and industry 4.0 (Bender et al., 2021; Madsen, 2019; Poethke et al., 2019). Work 4.0 refers to an increasingly digitalized, flexible, and boundaryless way of working, which has led to a reorganisation of work forms and conditions, influencing individual levels of collaboration as well as leadership and teamwork (Bender et al., 2021; Poethke et al., 2019). Industry 4.0, on the other hand, affects the manufacturing industry and is characterized by digital technologies, robotics, and artificial intelligence (Madsen, 2019), which have been further synthesized under the sub-dimension of *automation* below.

##### 3.2.2.1 ICTs

In the included literature, developments in ICTs were discussed as one of the most influential factors in the transformation of the working world (Georg et al., 2017; Labanauskaitė et al., 2021; Poethke et al., 2019). ICTs were found to affect almost all areas of work, leading to an increasing dependency on ICTs for the completion of tasks within companies (Poethke et al., 2019). Common forms of ICTs include the internet and smartphones (Poethke et al., 2019), work laptops (Fregnan et al., 2022), email, videoconferencing and instant messaging tools, and cloud-based platforms (Kinsman et al., 2024). As discussed in the previous section, the increasing use of these technologies in the work context has lead to greater temporal and spatial flexibility for employees (Adolph et al., 2016; Chen et al., 2023; Göllner and Rau, 2021; Hardering, 2021; Kinsman et al., 2024; Klinksiek et al., 2023; Poethke et al., 2019; Rödel and Krach, 2023; Zamani and Spanaki, 2023). Moreover, the use of digital technologies has been found to improve employee autonomy and connectivity, reduce commuting times and, if implemented well, provide a better work-life balance (Houghton et al., 2018; Rödel and Krach, 2023). Finally, Barbour et al. (2024) found that the adoption ICTS can enhance inclusion, allowing individuals with physical disabilities better access to the job market through more flexible working arrangements. However, ICTs also come with challenges. Besides physical factors like eyestrain (Khanwalkar and Dabir, 2022), a common critique discussed in the literature was the increased expectation of employee availability (see previous section on availability). As technology continues to dissolve boundaries, expectations of availability can become more and more severe (Rohwer et al., 2020).

##### 3.2.2.2 Virtual collaboration

Closely linked to the use of ICTs and different forms of flexibility are virtual teams, working together across spatial and temporal distances. Virtual collaboration describes new forms of collaboration between employees that are characterized by the use of ICTs (Akin and Rumpf, 2013). These technologies enable collaboration even when employees are not physically present in the same place (Schweitzer et al., 2020). It also helps companies to further a culture of innovation, which is needed as the global market changes and demands more flexibility from organizations (Akin and Rumpf, 2013).

The literature discussed many benefits associated with these changes. Virtual collaboration enables employees to be more autonomous in their work, which in turn facilitates self-organization (Hasenbein, 2021). This is also true on the company level, where teams can now collaborate even when they are not present in the same place, which has been an issue in large companies (Kühn et al., 2019). It also enables companies to draw from a large talent pool since they are no longer bound by geographical limitations (Edsall and Conrad, 2021). This also has the benefit of reducing the strain placed on infrastructure (Caldeira et al., 2023) and there has been evidence that this has lessened the exodus from rural areas (Schweitzer et al., 2020). However, virtual collaboration also comes with new challenges. One problem discussed in the included articles was that of interaction. Paradoxically, while ICTs enable more communication, researchers have found that virtual team members communicate less frequently (Afota et al., 2024). This might be because of the lack of spontaneous interaction in digital environments. While physical workspaces can lead to spontaneous social interaction, each interaction in digital spaces has to be planned and scheduled (Afota et al., 2024). Edsall and Conrad (2021) also found that feelings of belonging have reduced, and levels of loneliness increased, for people working digitally. Finally, for effective virtual collaboration to work, a robust technical infrastructure and a constant flow of information are essential (Figueira and da Costa, 2022).

##### 3.2.2.3 Automation

Finally, digitalization was also discussed in relation to increased levels of automation. The articles included in this review explored digitalization from various perspectives, with workplace automation and the use of artificial intelligence (AI) emerging as key topics (Klaser et al., 2023). Automation was mostly discussed within the context of manufacturing production and industry, leading to a reduction in costs for companies while increasing efficiency, flexibility, and product quality (Bayo-Moriones et al., 2015). Especially in industrial manufacturing, a shift from physical labor to monitoring robot-controlled processes was discussed (Rangraz and Pareto, 2021). However, articles also discussed that while automation offers potential savings in personnel costs, it also requires additional staff to monitor the automated processes (Confal et al., 2021). Automation processes in production are often based on a technology-centered approach, yet a human-centered design of workplaces is essential (Berretta et al., 2023). Berretta et al. (2023) concluded that despite increasing automation, the focus should be on designing a work environment that promotes motivation and identity and positively impacts the well-being of employees, which can be attained through further education (Rangraz and Pareto, 2021).

Automation was also discussed frequently in relation to the implementation of AI in various work processes (Poethke et al., 2019). In this context, AI encompassed a multitude of technologies, which can increase our understanding, analysis, and improvement of processes. In the literature, AI was considered essential for companies to remain competitive in today’s digitalized world (Rožman et al., 2023). For executives, AI was also an important tool for restructuring organizational frameworks (Rožman et al., 2023). However, the literature also discussed how digitalization and the increased use of AI are changing the labor market (Böhm and Stiglbauer, 2019). Automation was found to lead to the rationalization of workforce and an increase in automated jobs (Ivaldi et al., 2022; Rangraz and Pareto, 2021; Schermuly and Koch, 2019). This trend was particularly noticeable at low to medium skill levels and in the execution of routine tasks (Böhm and Stiglbauer, 2019; Confal et al., 2021; von Garrel and Düben, 2022). However, Confal et al. (2021) discussed that the introduction of AI could also allow more demanding activities to be replaced by technology in the future.

#### 3.2.3 Democratization

The conceptual framework of NW-settings also shared a common focus on democracy within companies (Lorra and Möltner, 2021; Schölmerich et al., 2023). This dimension of NW-settings refers to the effort of companies to address the complexity of the working world through structural changes in the form of less hierarchical organizational systems within the corporate framework. In this context, new forms of *autonomy*, *participation, leadership*, and *organizational structure* have been discussed in the literature (Kesselmann and Böhnke, 2021), forming the four sub-dimensions of democratization in the above presented model.

Overall, the included articles discussed how the transformation of workplace structures emphasized enhanced employee autonomy, participation in decision-making, and the erosion of traditional hierarchies (Bender et al., 2021; Poethke et al., 2019). Rather than eliminating organizational structures altogether, democratic workplaces typically aim to restructure them by promoting shared responsibility, integrating modern communication tools, and fostering cultures of learning, flexibility, and openness to failure (Kühn et al., 2019; Salmen and Festing, 2021). These cultural and structural shifts demand new forms of leadership aligned with decentralization and flexibility (Kesselmann and Böhnke, 2021; Soubelet-Fagoaga et al., 2021), underscoring the need for deliberate organizational changes to successfully implement democratic workplace structures.

##### 3.2.3.1 Autonomy

The conceptual framework of NW-settings emphasizes democratic workplace structures that prioritize autonomy as a fundamental aspect of work design (Lorra and Möltner, 2021; Rödel and Krach, 2023; Wendt, 2023). Autonomy is characterized by freedom and independence in task execution, fostering self-responsibility, initiative, and self-regulation (Arzenšek et al., 2021; Hasenbein, 2021; Niebuhr et al., 2022; Zirkler, 2023). It encompasses control over work sequencing and task execution underpinned by democratic decision-making, as well as free decision-making over work location and time (Barth and Blazejewski, 2023; Claassen et al., 2021; Kästner and Rudolph, 2022; Singe and Tietel, 2019). Furthermore, autonomy in determining work arrangements enhances employees’ ability to design their tasks, make independent decisions, and pursue innovative ideas (Camp et al., 2022; Schmitz et al., 2021). Empirical research has shown a positive correlation between autonomy and both, motivation and job satisfaction (Biemann and Weckmüller, 2015; von Garrel and Düben, 2022). In addition, it appeared that psychological empowerment, often associated with NW-settings, reinforces autonomy and self-determination, thereby expanding employees’ scope of action and responsibility while promoting health-supportive work conditions (Lorra and Möltner, 2021; Poethke et al., 2019; Stegh and Guthier, 2021; Schermuly and Koch, 2019). Additionally, autonomy extends to work scheduling and interaction, fostering team-based collaboration and reducing hierarchical constraints (Labanauskaitė et al., 2021; Scholl, 2020). However, research also highlighted that some individuals may resist greater autonomy due to personal preferences or organizational culture (Bachmann, 2022).

##### 3.2.3.2 Organizational structure

Organizational structure is another important sub-dimension of democratic workplaces, focusing on decentralization, participatory decision-making, and adaptive work processes. As work environments become increasingly digitized and networked, organizations are shifting towards more flexible and collaborative structures (Bender et al., 2021; Jochmaring and York, 2023). However, traditionally hierarchical companies often struggle to adapt to these changes, necessitating the implementation of NW-measures to foster democratized organizational structures (Lorra and Möltner, 2021). This transformation involves new collaboration models, role adaptations, and enhanced inter-organizational networking (Aroles et al., 2019; Bachmann and Quispe Bravo, 2021; Rödel and Krach, 2023). A key aspect of this shift was discussed as the flattening of hierarchies, facilitated by digital technologies that enabled decentralized decision-making and reduced managerial control (Bayo-Moriones et al., 2015; Confal et al., 2021). Empirical findings further highlight a trend towards participatory management, emphasizing shared decision-making and transparency (Claassen et al., 2021; Kühn et al., 2019). Self-management frameworks such as holacracy also distribute authority within organizations, replacing traditional managerial roles with collective intelligence and autonomous work structures (Ackermann et al., 2021; Schermuly and Koch, 2019; Zirkler, 2023).

##### 3.2.3.3 Participation

The included literature also highlights participation as a fundamental sub-dimension of democratic workplaces, emphasizing both collective co-determination and individual self-organization. Within the conceptual framework of NW-settings, participation is increasingly recognized as a necessary response to evolving work environments, requiring employees to engage more actively in decision-making processes (Graßmann and Decius, 2023; Rödel and Krach, 2023). Empirical evidence suggests that this shift is driven by both political imperatives for greater employee involvement and a growing desire among workers for meaningful co-determination in corporate decisions (Georg et al., 2017; Kühn et al., 2019; Poethke et al., 2019). As organizations move towards more democratic structures, participation encompasses direct engagement in change processes, transparency in decision-making, and an expansion of employees’ influence beyond traditional job autonomy (Biemann and Weckmüller, 2015; Labanauskaitė et al., 2021; Schlie and Wendland, 2023). Research further indicates that participative work environments facilitate problem-solving and foster a culture of shared responsibility (Bender et al., 2021; Biemann and Weckmüller, 2015; Göllner and Rau, 2021; Lorra and Möltner, 2021; Rangraz and Pareto, 2021). Participative management practices, such as self-managing teams, flexible office spaces, and democratic decision-making on collaboration, further enhance employees’ ability to influence their work environment (Barth and Blazejewski, 2023; Klinksiek et al., 2023; Singe and Tietel, 2019).

##### 3.2.3.4 Leadership

Finally, the included literature highlights the multifaceted nature of leadership in the context of NW-settings. While leadership styles and practices are not entirely novel, NW-settings demand a fundamental shift in leadership philosophy, emphasizing individualization, flexibility, collaboration, empowerment, and democracy (Klinksiek et al., 2023). A central goal of leadership in this framework was discussed as employee empowerment, reflected in less hierarchical structures and a shift from top-down to bottom-up approaches (Lorra and Möltner, 2021; Schermuly and Koch, 2019). The empirical evidence suggests that leadership in flat hierarchies requires managers to engage with employees on an equal basis, focusing on their development, and support self-organization (Schermuly and Koch, 2019; Scholl, 2020).

The empirical studies reviewed also discussed various leadership styles relevant to NW-settings, including shared leadership, in which leadership responsibilities are flexibly distributed based on expertise (Bachmann and Quispe Bravo, 2021; Scholl, 2020). Additionally, visionary, ambidextrous and transformational leadership are highlighted as crucial for fostering innovation, balancing creative exploration with structured goal-setting (Arzenšek et al., 2021; Figueira and da Costa, 2022; Gouda and Tiwari, 2024). Despite differences in approach, these leadership styles share common elements, particularly a tendency towards flat hierarchies and an emphasis on trust (Claassen et al., 2021; Kuzior et al., 2022). The literature indicates that trust is essential for virtual and cross-functional teams, helping to mitigate challenges such as uncertainty, social isolation, and digital disruptions (Gouda and Tiwari, 2024; Edsall and Conrad, 2021).

Furthermore, the reviewed studies identified key competencies required for leaders in NW-settings, including social and communication skills (Kühn et al., 2019; Labanauskaitė et al., 2021), the ability to provide constructive feedback (Rödel and Krach, 2023; Schmitz et al., 2021), and self-regulation (Geldart, 2022). Leadership in NW-settings also requires digital literacy, vision, and a role model function to navigate the complexities of modern work environments (Claassen et al., 2021). Overall, the literature suggests that effective leadership in democratic workplaces is characterized by participatory, trust-based, and adaptable approaches that foster employee engagement and organizational resilience.

#### 3.2.4 Agility

The dimension of agility, which was synthesized as the final dimension of NW-settings, has considerable overlap with the dimensions of flexibility, digitalization and democratization (Coban and Wenten, 2021; Ivaldi et al., 2022; Kühn et al., 2019; Labanauskaitė et al., 2021), since it includes aspects of flexible work arrangements, technological changes, and working in democratic workplaces. However, agility has a distinct focus on both responsiveness in relation to market changes (Gouda and Tiwari, 2024) and agile workforces who use modern tools to foster an environment of fast innovation (Bachmann and Quispe Bravo, 2021). In the current business environment, companies need to cope with rapid changes in the market (Fregnan et al., 2022; Gouda and Tiwari, 2024). This requires thinking outside the box (Fregnan et al., 2022) and fostering a more individualized and effective work environment (Klaser et al., 2023). To achieve this, companies have been found to rely more on self-organized work (Kreyenberg, 2023). In addition, product cycles are changing in response to new market demands and are becoming faster (Ötting et al., 2021). To keep up with the demands for innovation, *agile methods* are becoming more common, such as brainstorming sessions with interdisciplinary participants (Coban and Wenten, 2021) or design thinking workshops (Rödel and Krach, 2023). In addition, the aspects of *proactivity* and *reactivity* in the context of agile work settings was discussed in the literature in relation to the way companies are responding to the potential of external market changes.

Many articles discussed the positive aspects of agility, especially when it comes to fostering psychological empowerment in employees (Graßmann and Decius, 2023; Klinksiek et al., 2023; Schermuly and Koch, 2019). However, these agile work environments can also lead to new challenges for employees (Bachmann, 2022). There has been an acceleration when it comes to the demands and changes in a workplace (Bachmann, 2022), which can drive work intensity and job insecurity (Confal et al., 2021). To combat this, Bachmann (2022) argued that companies need to commit to new ways of working and not only try to make incremental changes towards agile organization.

##### 3.2.4.1 Agile methods

According to the included literature, agile methods encompassed the reorganization of structures and processes within companies (Schneider, 2020). Common techniques for this are kanban, scrum, and design thinking workshops, to name just a few (Bachmann and Quispe Bravo, 2021; Hasenbein, 2021; Rödel and Krach, 2023). The scrum board, for example, has the purpose of transparently displaying what is being worked on at any given time. This has become increasingly necessary to keep track of a variety of different tasks in fast, iterative work processes (Coban and Wenten, 2021). These methods are part of what has been called an ‘agile mindset’, which works best when combined with a holocratic organization of work (Ackermann et al., 2021). This is to further collaboration, trust, and knowledge sharing in an organization (Bachmann and Quispe Bravo, 2021), in order to replace outdated depersonalized processes with agile and flexible ones to stay competitive in modern markets and to further support the health of employees (Coban and Wenten, 2021; Schneider, 2020).

##### 3.2.4.2 Proactivity

According to Ivaldi et al. (2022), a proactive company adopts a culture of learning to not only cope with changes in the business environment but also to anticipate them. To achieve this, companies need to be open to change and support new ideas in relation to work arrangements and leadership styles (Barth and Blazejewski, 2023). However, it has also been noted that proactivity does not only come from the company but also from the workforce itself. Employees must increasingly adapt to faster changes. To achieve this, the literature argued that companies should foster a climate of openness, where employees are incentivized to always keep learning through work (Graßmann and Decius, 2023). In the specific context of manufacturing and production, proactivity can also be achieved through different processes, such as rapid prototyping, which incentivizes building prototypes in the early stages of development. This can boost efficiency and give developers more chances to experiment (Coban and Wenten, 2021).

##### 3.2.4.3 Reactivity

In contrast to proactivity, reactivity was described as the capacity of a company to adapt to and even take advantage of changes in the market environment (Salmen and Festing, 2021). To achieve this, Ivaldi et al. (2022) discussed that companies need to create an environment that is responsive to internal and external stimuli and where knowledge is openly shared. Collective reflections of the workforce on used business strategies and production processes can also be a part of this and help companies avoid repeating mistakes (Georg et al., 2017). The reactivity of many companies was particularly discussed in the context of the COVID-19 pandemic (Caldeira et al., 2023). Companies had to adapt rapidly to external changes, embracing new work arrangements and technologies (Fregnan et al., 2022). In addition, sustainability has been discussed as an important part of reactivity. To be sustainable, companies need to put individual needs and social changes at the center of attention when they are adapting work systems and processes (Kuzior et al., 2022). The term humanistic management was used to describe how companies apply sustainability within their organization. It emphasizes sustainable human development, which focuses on a positive impact on employees (Kuzior et al., 2022).

## 4 Discussion

The aim of this systematic scoping review was to map out the existing literature and to develop an integrated conceptual framework of NW-settings. Based on the included literature, an integrated conceptual framework of NW-settings was developed, which consists of four main dimensions: flexibility, digitalization, democratization and agility, as well as 14 inter-related sub-dimensions. As the synthesis of the NW-settings framework showed, however, these dimensions and sub-dimensions often overlapped and did not have clear conceptual boundaries. In addition, the significance of the dimensions appeared to occur along a continuum, with workplaces implementing different levels of NW-measures.

Overall, the scoping review revealed that most research focused on the perspective of employees/workers with a particular focus on remote and digital workers, as well as on employers/managers, from a broad range of industries. Due to the search terms focusing on NW and work 4.0, two terms that are mostly used in German-speaking countries, the majority of included articles were published in Germany. Nonetheless, the review also incorporated international studies from over 21 countries, with the majority originating from Western contexts, particularly across Europe and North America.

### 4.1 Strengths and limitations

A major strength of this systematic scoping review was its rigorous and systematic approach, which adhered to the established frameworks of Arksey and O'malley (2005) and Peters et al. (2015a). This methodological rigor ensured transparency and reproducibility throughout the review process (Peters et al., 2015b). Furthermore, a comprehensive search strategy was employed, with a focus on well-defined search terms, which contributes to the robustness of the findings.

Despite its strengths, this review has several limitations. First, the search string primarily utilized the term “New Work,” and “Work 4.0,” thereby putting less focus on synonymous expressions such as “New Ways of Working,” “Modern Work” or “New forms of Work.” This decision was made to reflect the specific project context and the predominance of the term in German-speaking regions; however, the use of diverse and comprehensive search terms in relation to different NW-measures (e.g., agility, flexibility, empowerment, work-life-balance, leadership, participation, digitalization, and automation) may have mitigated concerns regarding generalizability. In addition, while the review was rooted in the German context, it integrated various global perspectives, reflecting transnational trends in employment transformations and vast organizational changes due to increased digitalization, flexibilization and democratization processes of work.

Second, due to time and resource constraints, the review included only academic articles in English and German, omitting grey literature and studies in other languages. This may have resulted in the exclusion of potentially relevant government guidelines and organizational strategies. Finally, consistent with the nature of scoping reviews (Munn et al., 2018; Peters et al., 2015b), no formal quality appraisal of the included studies was conducted. Consequently, findings from methodologically weaker studies may have been included, warranting cautious interpretation (Hong and Pluye, 2019). Future research could address these limitations by broadening the search terminology, incorporating grey literature, and including studies in additional languages.

### 4.2 Implications for practice and further research

This scoping review contributes to the comprehensive literature on New Work (NW) by offering an integrated conceptual framework that synthesizes current literature and expert perspectives. Given that NW is often used as a broad and ambiguous “container term,” this framework provides a more structured and comprehensive definition of NW-settings. By delineating key dimensions - flexibility, digitalization, democratization, and agility - the framework facilitates a clearer understanding of NW and serves as a foundation for further empirical investigations. It is important to note, however, that the underpinning dimensions of NW-settings identified in this review are not new—on the contrary, they have been implemented in practice for some time now. However, their combination and mutual reinforcement define the character of contemporary NW-settings and reflect the increasing complexity of organizational responses to the changing world of work.

Contrary to the common belief that NW is predominantly implemented in technology and knowledge-intensive industry, this review highlighted that NW-settings are present across all different industries, suggesting that the different NW dimensions - flexibility, digitalization, democratization, and agility - can be adapted to and benefit organizations in traditional industries as well. In today’s world of NW, the interdependence and interaction of the four NW-settings is central. These elements have a complex relationship with employee participation and influence both internal structures and higher levels of management. Project work is one form of individualization, particularly in an era of variable working practices that require individual adaptability in terms of place, time and contract design. This raises the critical question of the extent to which a return to collective forms of work makes sense in an increasingly flexible working environment.

The conceptual framework also lays the foundation for rethinking workplace transformation at the intersection of organizational innovation and social responsibility. The future of work should be viewed not only through a lens of efficiency or competitiveness, but also in terms of inclusion, ethics, and sustainability. The socio-cultural context in which NW is theorized and applied plays a critical role in shaping its implementation and outcomes. While this scoping review integrates both German and international perspectives, NW-settings are inevitably shaped by the socio-cultural context in which they have most actively been theorized and implemented. As such, the applicability of the conceptual framework of NW-settings developed in this review, which focused predominantly on the German and Western context, may vary in societies with different institutional structures, ethical standards, and labor market norms. Future research should explore how NW-settings are locally adapted and negotiated across diverse cultural settings.

In practical terms, the conceptual framework of NW-settings has been pivotal for the *BGM4NewWork* project, where it was further validated through qualitative interviews and factor analyses, and subsequently applied in a quantitative survey to operationalize the different dimensions of NW. The findings from this research project will inform concrete recommendations on how OHM should be adapted to better address the specific health needs of employees working in NW-settings. Given the increasing adoption of NW principles in organizations, these insights will be valuable for employers, policymakers, and health professionals in designing work environments that support employee health and well-being. In particular, future occupational health research on NW should focus on empirically investigating the effects of participative approaches to shed light on the balance between flexibility and employees’ health. Practical implications for OHM and the sustainable implementation of NW can be derived from a critical examination of these aspects. Ultimately, a harmonized integration of the four NW-settings dimensions is required to meet the challenges of the modern working world and to ensure employee satisfaction and performance in the long term. Future research should analyze which components are part of best practice regarding participative and democratic approaches in modern workplaces.

Looking ahead, future research should thus investigate how organizations can implement NW in a way that fosters employee participation, protects workers’ rights, and supports well-being in an ethically sound manner. In this context, it is particularly important to capture the voices of different stakeholders, including employees, managers, HR, and health actors (such as doctors and medical decision-makers). In addition, human rights aspects must be considered to ensure that working conditions are fair and non-discriminatory. Protecting workers’ rights should be seen not only as a legal obligation, but also as an ethical concern that must be at the heart of business. Such a comprehensive analysis could help to develop a nuanced understanding of how different perspectives and needs can be integrated into the design of work environments. Employees can provide valuable insights into the real demands and challenges of everyday work, while managers have a crucial role to play in implementing and supporting participative structures. Healthcare professionals bring important professional perspectives, particularly about the well-being and mental health of workers. Their expertise is crucial in developing appropriate measures to support employees’ performance, health maintenance and general well-being. By including these different perspectives in the discourse, companies can develop innovative solutions that meet the needs of their employees and promote a corporate culture that supports sustainable development. Ultimately, such an inclusive approach could help to create a work-friendly environment that improves both the health and satisfaction of employees and the overall performance of the organization.

Finally, the development of standardized measurement instruments for the dimensions of NW-settings could facilitate more rigorous evaluations and support the evolution of a cohesive body of knowledge that bridges theoretical perspectives with empirical evidence. Interdisciplinary research combining perspectives from sociology, organizational psychology, occupational health, and digital transformation studies could provide a more holistic understanding of how NW can be effectively implemented. Only by embracing this multifaceted approach can we shape the future of work in ways that are innovative, inclusive, and sustainable for individuals, organizations, and society.

### 4.3 Conclusion

In conclusion, this systematic scoping review has yielded an integrated conceptual framework for NW-settings that encapsulates the key dimensions of flexibility, digitalization, democratization, and agility. Overall, the significance of the dimensions appeared to occur along a continuum, with workplaces implementing different levels of NW-measures, i.e., a workplace may have implemented NW-measures across all four dimensions or focus on just one sub-dimension and were still considered a NW-setting in this review. In addition, the synthesis of the NW-settings framework showed that the dimensions of NW-settings and the 14 sub-dimensions often overlapped and did not have clear conceptual boundaries.

This scoping review addressed a critical gap in the literature by systematically synthesizing the fragmented literature on NW, offering a clear conceptualization of NW-settings to guide future empirical studies and practical interventions. By focusing on the environmental and structural conditions in which NW is implemented, this review helped distinguish between abstract NW ideals and their real-world applications in organizational settings, which have been implemented across a range of different work environments. Thus, this review highlighted that NW is not a singular, abstract ideal but a multidimensional and context-dependent organizational reality. As our world of work continues to evolve, we hope that the conceptual framework of NW-settings developed in this review offers a critical tool for understanding how emerging work models can be designed and governed to balance organizational transformation with individual well-being and social equity in diverse labor market contexts.
