# Supplementary material for: Developing an integrated conceptual framework of NewWork-settings: a systematic scoping review
Source: Front Sociol. 2025 Sep 30;10:1631523. doi: 10.3389/fsoc.2025.1631523 (PMC12519458; doi:10.3389/fsoc.2025.1631523)
Supplement: Supplementary file 1 [file Table_1.pdf]

Supplementary Table 1: Characteristics of included articles

	N = 99	Included articles
**Publication type**		
Quantitative Research (RCTs, cohort studies, etc.)	33	(Afota et al., 2024); (Akin and Rumpf, 2013); (Bachmann and Quispe Bravo, 2021); (Barbour et al., 2024); (Becker et al., 2020); (Bender et al., 2022); (Berretta et al., 2023); (Chen et al., 2023); (Claassen et al., 2021); (Confal et al., 2021); (Dolce et al., 2020); (Ekpanyaskul et al., 2023); (Ekpanyaskul and Padungtod, 2021); (Florin and Pichault, 2020); (Graßmann and Decius, 2023);(Kästner and Rudolph, 2022); (Kesselmann and Böhnke, 2021); (Khanwalkar and Dabir, 2022); (Kortsch et al., 2022); (Klaser et al., 2023); (Lorra and Möltner, 2021); (Niebuhr et al., 2022); (Parent-Lamarche and Laforce, 2022); (Parker and Knight, 2024); (Poethke et al., 2019); (Rohwer et al., 2020); (Rožman et al., 2023); (Schmitz et al., 2021); (Schlie and Wendland, 2023); (Schölmerich et al., 2023); (Smite et al., 2023); (von Garrel and Düben, 2022); (Wong et al., 2020)
Overview	21	(Adolph et al., 2016); (Braml, 2022); (Christensen, 2023); (Fuchs and Cumbers, 2023); (Geldart, 2022); (Georgi, 2021); (Gouda and Tiwari, 2024); (Hasenbein, 2021); (Jäckel, 2020); (Jochmaring and York, 2023); (Kreyenberg, 2023); (Rödel and Krach, 2023); (Schermuly, 2019); (Scholl, 2020); (Singe and Tietel, 2019); (Stecker and Kionke, 2020); (Stegh and Guthier, 2021); (Terry, 2022); (Wendt, 2023); (Zamani and Spanaki, 2023); (Zirkler, 2023)
Qualitative Research (Case study, ethnography, etc.)	20	(Ackermann et al., 2021); (Baba et al., 2021); (Bachmann, 2022); (Barth and Blazejewski, 2023); (Bayo-Moriones et al., 2015); (Coban and Wenten, 2021); (Degen and Zekavat, 2022); (Deng and Joshi, 2016); (Georg et al., 2017); (Houghton et al., 2018); (Ivaldi et al., 2022); (Kühn et al., 2019); (Kuzior et al., 2022); (Lin and Wang, 2022); (Madsen, 2019); (Ötting et al., 2021); (Petrakova et al., 2021); (Rangraz and Pareto, 2021); (Schneider, 2020); (Schweitzer et al., 2020)
Review	13	(Aroles et al., 2019); (Biemann and Weckmüller, 2015); (Camp et al., 2022); (Figueira and da Costa, 2022); (Hardering, 2021); (Kinsman et al., 2024); (Klinksiek et al., 2023); (Popescu et

		al., 2020); (Salmen and Festing, 2021); (Santa and Popescu, 2021); (Schermuly and Koch, 2019); (Strikovic and Wittmann, 2022); (Varma et al., 2022)
Mixed-Methods	12	(Arzenšek et al., 2021); (Böhm and Stiglbauer, 2019); (Caldeira et al., 2023); (Edsall and Conrad, 2021); (Fregnan et al., 2022); (Göllner and Rau, 2021); (Kossek and Ollier-Malaterre, 2020); (Labanauskaitė et al., 2021); (Nicolaisen, 2014); (Silva Júnior et al., 2022); (Soubelet-Fagoaga et al., 2021); (Stoian et al., 2022)
**NW-Populations, where reported**		
Workers/Employees (not further specified)	20	(Ackermann et al., 2021); (Arzenšek et al., 2021); (Barth and Blazejewski, 2023); (Bender et al., 2022); (Chen et al., 2023); (Ekpanyaskul and Padungtod, 2021); (Fregnan et al., 2022); (Göllner and Rau, 2021); (Houghton et al., 2018); (Kortsch et al., 2022); (Kühn et al., 2019); (Labanauskaitė et al., 2021); (Parent-Lamarche and Laforce, 2022); (Parker and Knight, 2024); (Rangraz and Pareto, 2021); (Schlie and Wendland, 2023); (Schölmerich et al., 2023); (Smite et al., 2023); (Soubelet-Fagoaga et al., 2021); (Stoian et al., 2022)
Managers/employers	19	(Akin and Rumpf, 2013); (Barth and Blazejewski, 2023); (Bayo-Moriones et al., 2015); (Degen and Zekavat, 2022); (Fregnan et al., 2022); (Gouda and Tiwari, 2024); (Graßmann and Decius, 2023); (Kossek and Ollier-Malaterre, 2020); (Labanauskaitė et al., 2021); (Nicolaisen, 2014); (Ötting et al., 2021); (Parker and Knight, 2024); (Rangraz and Pareto, 2021); (Rödel and Krach, 2023); (Rožman et al., 2023); (Schlie and Wendland, 2023); (Schölmerich et al., 2023); (Stoian et al., 2022); (Wendt, 2023)
Remote workers, teleworkers (virtual team members, high-intensity telecommuters, coworking space workers)	11	(Afota et al., 2024); (Aroles et al., 2019); (Edsall and Conrad, 2021); (Ekpanyaskul et al., 2023); (Khanwalkar and Dabir, 2022); (Klaser et al., 2023); (Kinsman et al., 2024); (Niebuhr et al., 2022); (Rohwer et al., 2020); (Soubelet-Fagoaga et al., 2021); (Zamani and Spanaki, 2023)

Technology based workers (e.g. technology developers, software engineers) and digital labor (e.g. digital nomads, clickworkers, crowdworkers, YouTube Creators, app-based drivers)	10	(Afota et al., 2024); (Aroles et al., 2019); (Bachmann and Quispe Bravo, 2021); (Caldeira et al., 2023); (Claassen et al., 2021); (Coban and Wenten, 2021); (Confal et al., 2021); (Deng and Joshi, 2016); (Silva Júnior et al., 2022); (Wong et al., 2020)
Company related (HR, administration, work council, operations)	7	(Afota et al., 2024); (Bachmann and Quispe Bravo, 2021); (Barth and Blazejewski, 2023); (Georg et al., 2017); (Klaser et al., 2023); (Kossek and Ollier-Malaterre, 2020); (Ötting et al., 2021)
Knowledge workers (skilled workers, white collar workers, high potentials)	5	(Bachmann, 2022); (Kuzior et al., 2022); (Schweitzer et al., 2020); (von Garrel and Düben, 2022); (Zamani and Spanaki, 2023)
Self-employed	4	(Aroles et al., 2019); (Arzenšek et al., 2021); (Florin and Pichault, 2020); (Fregnan et al., 2022)
Others (Customer service, operators, teachers, working students, workers with disabilities)	7	(Baba et al., 2021); (Fregnan et al., 2022); (Jochmaring and York, 2023); (Klinksiek et al., 2023); (Petrakova et al., 2021); (Rangraz and Pareto, 2021); (Schmitz et al., 2021)
**NW-Industries, where reported**		
Manufacturing (e.g. automotive, chemistry and pharmaceutical,	15	(Ackermann et al., 2021); (Akin and Rumpf, 2013); (Bayo-Moriones et al., 2015); (Dolce et al., 2020)<br>(Fregnan et al., 2022); (Georg et al., 2017); (Kesselmann and Böhnke, 2021); (Kossek and Ollier-Malaterre, 2020); (Niebuhr et al., 2022); (Ötting et al., 2021); (Poethke et al., 2019);

metal- and electronics, mechanical engineering)		(Popescu et al., 2020); (Rožman et al., 2023); (Salmen and Festing, 2021); (Schölmerich et al., 2023)
Banking and insurance	14	(Afota et al., 2024); (Akin and Rumpf, 2013); (Barth and Blazejewski, 2023); (Kästner and Rudolph, 2022); (Kinsman et al., 2024); (Kortsch et al., 2022); (Kossek and Ollier-Malaterre, 2020);<br>(Kühn et al., 2019); (Nicolaisen, 2014); (Parker and Knight, 2024); (Poethke et al., 2019); (Rožman et al., 2023); (Schlie and Wendland, 2023);(Schölmerich et al., 2023)
IT/ Software Development	13	(Akin and Rumpf, 2013); (Bachmann and Quispe Bravo, 2021); (Confal et al., 2021); (Göllner and Rau, 2021); (Kinsman et al., 2024); (Kossek and Ollier-Malaterre, 2020); (Kuzior et al., 2022); (Lorra and Möltner, 2021); (Rohwer et al., 2020); (Salmen and Festing, 2021); (Schneider, 2020); (Schölmerich et al., 2023); (Smite et al., 2023)
Public sector (e.g. government, education and research)	13	(Bachmann and Quispe Bravo, 2021); (Claassen et al., 2021); (Dolce et al., 2020); (Houghton et al., 2018); (Kesselmann and Böhnke, 2021); (Kinsman et al., 2024); (Lorra and Möltner, 2021); (Parker and Knight, 2024); (Petrakova et al., 2021); (Poethke et al., 2019); (Rožman et al., 2023); (Salmen and Festing, 2021); (Schmitz et al., 2021)
Healthcare and Social Work	9	(Bachmann and Quispe Bravo, 2021); (Berretta et al., 2023); (Böhm and Stiglbauer, 2019); (Lorra and Möltner, 2021); (Niebuhr et al., 2022); (Parent-Lamarche and Laforce, 2022); (Poethke et al., 2019); (Rožman et al., 2023); (Salmen and Festing, 2021)
Creative industry and communication	7	(Akin and Rumpf, 2013); (Dolce et al., 2020); (Graßmann and Decius, 2023); (Kinsman et al., 2024); (Parker and Knight, 2024); (Rožman et al., 2023); (Salmen and Festing, 2021)
Service sector (e.g. support services)	6	(Bachmann and Quispe Bravo, 2021); (Dolce et al., 2020); (Kesselmann and Böhnke, 2021); (Lorra and Möltner, 2021); (Rožman et al., 2023); (Salmen and Festing, 2021)
Consulting	6	(Böhm and Stiglbauer, 2019); (Kinsman et al., 2024); (Kossek and Ollier-Malaterre, 2020); (Lorra and Möltner, 2021); (Poethke et al., 2019); (Schölmerich et al., 2023)

Associations, institutions, and NGOs	3	(Bachmann and Quispe Bravo, 2021); (Lorra and Möltner, 2021); (Parker and Knight, 2024)
Tourism	3	(Kinsman et al., 2024); (Kossek and Ollier-Malaterre, 2020); (Poethke et al., 2019)
Crowdsourcing (e.g. MTurk)	3	(Deng and Joshi, 2016); (Parker and Knight, 2024); (Wong et al., 2020)
Other (e.g. real estate, farming, energy, transport)	4	(Göllner and Rau, 2021); (Kästner and Rudolph, 2022); (Parker and Knight, 2024); (Rožman et al., 2023)
**Regions and Countries**		
**German-speaking countries** (Austria, Germany, Switzerland)	53	(Adolph et al., 2016); (Akin and Rumpf, 2013); (Bachmann, 2022); (Bachmann and Quispe Bravo, 2021); (Becker et al., 2020); (Bender et al., 2022); (Berretta et al., 2023); (Biemann and Weckmüller, 2015); (Böhm and Stiglbauer, 2019); (Braml, 2022); (Christensen, 2023); (Claassen et al., 2021); (Coban and Wenten, 2021); (Confal et al., 2021); (Degen and Zekavat, 2022); (Fuchs and Cumbers, 2023); (Geldart, 2022); (Georg et al., 2017); (Georgi, 2021); (Graßmann and Decius, 2023); (Göllner and Rau, 2021); (Hardering, 2021); (Hasenbein, 2021); (Jäckel, 2020); (Jochmaring and York, 2023); (Kästner and Rudolph, 2022); (Kesselmann and Böhnke, 2021); (Kreyenberg, 2023); (Kortsch et al., 2022); (Kühn et al., 2019); (Lorra and Möltner, 2021); (Madsen, 2019); (Niebuhr et al., 2022); (Ötting et al., 2021); (Poethke et al., 2019); (Popescu et al., 2020); (Rohwer et al., 2020); (Rödel and Krach, 2023); (Salmen and Festing, 2021); (Schermuly, 2019); (Schermuly and Koch, 2019); (Schlie and Wendland, 2023); (Schmitz et al., 2021); (Schneider, 2020); (Scholl, 2020); (Schölmerich et al., 2023); (Schweitzer et al., 2020); (Singe and Tietel, 2019); (Stecker and Kionke, 2020); (Stegh and Guthier, 2021); (von Garrel and Düben, 2022); (Wendt, 2023); (Zirkler, 2023)
**Europe** (Belgium, France, Italy, Norway, Netherlands, Poland, Romania, Russia,	26	(Ackermann et al., 2021); (Afota et al., 2024); (Aroles et al., 2019); (Arzenšek et al., 2021); (Barth and Blazejewski, 2023); (Bayo-Moriones et al., 2015); (Caldeira et al., 2023); (Dolce et al., 2020); (Florin and Pichault, 2020); (Fregnan et al., 2022); (Gouda and Tiwari, 2024); (Ivaldi et al., 2022); (Klaser et al., 2023); (Klinksiek et al., 2023); (Kossek and Ollier-Malaterre, 2020);

Slovenia, Sweden, Spain, UK, Lithuania)		(Kuzior et al., 2022); (Labanauskaitė et al., 2021); (Nicolaisen, 2014); (Petrakova et al., 2021); (Rangraz and Pareto, 2021); (Santa and Popescu, 2021); (Soubelet-Fagoaga et al., 2021); (Stoian et al., 2022); (Varma et al., 2022); (Wong et al., 2020); (Zamani and Spanaki, 2023)
**North America** (Canada, USA)	11	(Baba et al., 2021); (Barbour et al., 2024); (Camp et al., 2022); (Chen et al., 2023); (Deng and Joshi, 2016); (Edsall and Conrad, 2021); (Parent-Lamarche and Laforce, 2022); (Rožman et al., 2023); (Smite et al., 2023); (Strikovic and Wittmann, 2022); (Terry, 2022)
**Asia** (India, Japan, South Korea, Taiwan)	4	(Ekpanyaskul et al., 2023); (Ekpanyaskul and Padungtod, 2021) ; (Khanwalkar and Dabir, 2022); (Lin and Wang, 2022)
**Australia and Oceania** (Australia)	3	(Houghton et al., 2018); (Kinsman et al., 2024); (Parker and Knight, 2024)
**South America** (Brazil)	2	(Figueira and da Costa, 2022); (Silva Júnior et al., 2022)
